# Catheter Ablation of Atrial Fibrillation: Technique and Future Perspectives

**DOI:** 10.3390/jcm14061788

**Published:** 2025-03-07

**Authors:** Francesco Peruzza, Andrea Candelora, Carlo Angheben, Massimiliano Maines, Mauro Laurente, Domenico Catanzariti, Maurizio Del Greco, Antonio Madaffari

**Affiliations:** 1 Department of Cardiology, Santa Maria del Carmine Hospital, Corso Verona 4, 38068 Rovereto, Italy; francesco.peruzza@apss.tn.it (F.P.); massimiliano.maines@apss.tn.it (M.M.); mauro.laurente@apss.tn.it (M.L.); maurizio.delgreco@apss.tn.it (M.D.G.); 2 Azienda Provinciale per i Servizi Socio Sanitari–APSS, 38123 Trento, Italy

**Keywords:** atrial fibrillation, catheter ablation, pulsed field, pulmonary vein isolation, posterior wall isolation, vein of Marshall

## Abstract

Atrial fibrillation is the most common sustained cardiac arrhythmia with a significant impact on quality of life in terms of symptoms and reduction of functional status. Also, it is associated with an increased risk of mortality, stroke, and peripheral embolism. Catheter ablation for atrial fibrillation has become a well-established treatment, improving arrhythmia outcomes without increasing the risk of serious adverse events compared to antiarrhythmic drug therapy. The field has undergone significant advancements in recent years, yet pulmonary vein isolation continues to be the cornerstone of any atrial fibrillation ablation procedure. The purpose of this review is to provide an overview of the current techniques, emerging technologies, and future directions.

## 1. Introduction

Atrial fibrillation (AF) is the most common supraventricular arrhythmia, characterized by chaotic atrial electrical activation leading to ineffective atrial contraction. Patients with AF may experience different types of symptoms such as palpitations, dyspnea, dizziness, fatigue, pre-syncope, and syncope, with high interpatient variability. These symptoms have a remarkable impact on quality of life (QoL) [1].

Moreover, patients with AF have an increased risk of mortality, stroke, peripheral embolism, and heart failure relapses [2,3].

An early rhythm control strategy is associated with improved cardiovascular outcomes. Antiarrhythmic drugs (AADs) have a limited efficacy in preventing AF recurrences with significant adverse event rates [4]. Conversely, catheter ablation (CA) has become an evidence-based, safe, and powerful treatment, even as first-line therapy. Several multicenter randomized clinical trials (RCTs) have demonstrated superiority of CA over AADs in reducing AF recurrences and progression of the disease, improving symptoms, and QoL [5,6,7,8]. The primary approach to ablation involves the isolation of pulmonary veins (PVI) from the left atrium (LA). The PVI approach has demonstrated success rates ranging from 60% to 90% at 12-month follow-up in patients with paroxysmal AF (PAF). However, in persistent atrial fibrillation (PsAF), post-ablation outcomes are significantly less favorable, often requiring repeat procedures. Non-PV triggers may contribute o AF maintenance. Strategies combining PVI with extra-PV substrate ablation, combined with imaging information, have been tested to improve CA efficacy, with mixed results. This review will delve into the various ablation techniques and energy sources used (with a focus on the emerging energy), the role of imaging, the targets of ablation (both PV and extra-PV triggers), and future directions in this field.

## 2. Brief Historical Overview

Until the 90s, the cornerstones of AF treatment were stroke risk modification with anticoagulation therapy, attempts to interrupt and/or prevent AF recurrences with AADs or cardioversion, and rate management (medication or “ablate and pace” strategy).

The dawn of a new therapeutic option was following a description of the surgical MAZE procedure by Cox and coll. [9], based on a transmural atrial incision in order to reduce the ability of the atria to fibrillate. The main limitation of these techniques is the need for cardiopulmonary bypass and the implicit surgical risk. However, the concept of atria partition in several segments, no longer able to sustain AF, was used in the first ablation attempts with linear lesions [10].

In 1998, Haissaguerre et al. first described in his pioneering work premature atrial contractions from the pulmonary veins (PVs) as AF drivers in patients with paroxysmal AF (PAF) [11]. Today, pulmonary vein isolation (PVI) remains the cornerstone of any AF interventional treatment.

Over the years, new ablation strategies and technological innovations have been introduced to enhance the safety, efficiency, and efficacy of AF ablation.

## 3. Transcatheter Ablation for Atrial Fibrillation: Techniques and Energy Sources

The initial technique for PVI included a focal ablation inside the PVs, with a significantly increased risk of PV stenosis [10,12]. Further, this approach was also characterized by multiple cardioversions during the procedure because of the need for AF induction for trigger mapping [13]. The field then moved from a segmental ostial to a wide circumferential PVI, ultimately verifying conduction block [14,15,16]. A randomized study demonstrated the superiority of an antral approach with respect to the ostial segmental isolation [17], also resulting in lower rates of PV stenosis. Antral PVI is now widely accepted as the goal of AF ablation techniques and is recommended for all AF ablation procedures (class I indication, level of evidence A) [18]. In patients with PAF, antral PVI is associated with a freedom from AF recurrence ranging from 60% to 79%, with long term freedom reaching up to 77% [5,19]. This approach can be performed through a point-by-point or a one-shot technique. The advent of three-dimensional electroanatomic mapping (3D-EAM) over fluoroscopy dramatically changed the field.

The main energy forms to obtain antral PVI can be divided into thermal (radiofrequency, cryoablation, laser) and non-thermal energy (electroporation, ethanol).

### 3.1. Radiofrequency Ablation vs. Cryoballoon Ablation

Radiofrequency ablation (RFA) is the most widely used technique. It is based on high-frequency (500–1000 kHz) electrical energy to generate heat, which is delivered through a catheter tip to the target tissue. The heat causes thermal injury, leading to the formation of scar tissue that interrupts electrical conduction. RFA, usually performed with irrigated-tip catheters, is mostly, but not only, delivered with a point-by-point approach with a power of 20 to 40 W for 20 to 40 s.

Several studies have explored the use of markers for assessing ablation lesion quality alongside 3D-EAM, such as the ablation index (AI) and lesion size (LSI). The AI is a composite measure that includes power, force, stability, and duration, while the LSI combines contact force, ablation time, and the amount of energy delivered [20,21].

In 2018, Taghji et al. described the CLOSE protocol for PVI that considers the AI (a value ≥ 400 at the posterior wall and ≥ 550 at the anterior wall), interlesion distance (≤ 6 mm), and catheter stability [22]. Subsequently, Duytschaever et al. published the CLOSE to CURE study in which 105 patients with PAF undergoing the CLOSE ablation protocol had freedom from any atrial arrhythmia 87% at 1 year and 78% at 2 years after a single procedure. The follow-up was performed with an implantable loop recorder, and after PVI (1.13 ± 0.39 procedures per patient), the median atrial arrhythmia burden decreased to 0% at the 1-year and 2-year follow-ups [23].

**Figure 1:**
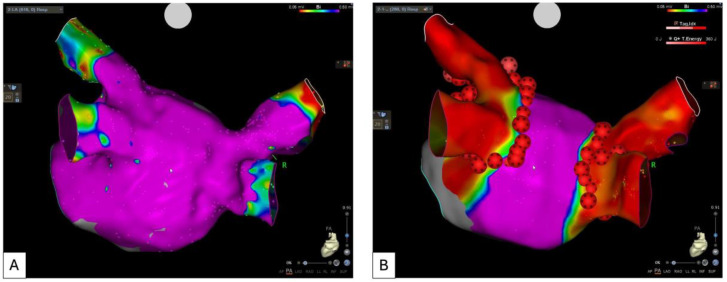
Three-dimensional left atrial electroanatomic mapping with an example of pre and post pulmonary veins isolation (respectively panel **A** and **B**) with a very high power-short duration strategy (CARTO System—90 watts for 4 seconds).

**Figure 2:**
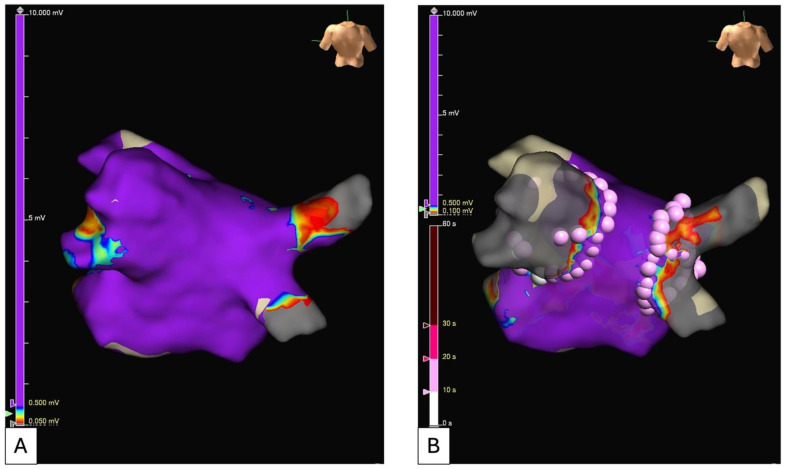
An example of three-dimensional electroanatomic mapping showing pre (panel **A**) and post (panel **B**) ablation with high power-short duration (ENSITE system—50 watts for 10 s).

Recently, a high power and short duration strategy has been evaluated for RFA (Figure 1 and Figure 2). This protocol consists of delivering a power from 50 to 90 W for four to ten seconds, respectively, high power and very high power, in order to create a more reliable and irreversible lesion size, minimizing damage related to time-dependent conductive heating [24].

Cryoballoon ablation (CBA) consists of an “over the wire” balloon catheter that is deployed at the PV antrum. The balloon is inflated and cooled with nitrous oxide (NO), and lesion formation occurs through convective cooling, which absorbs heat from the myocardium, leading to ischemic cell death via ice crystal formation. The current protocol requires a single application if the temperature reaches −40° and acute electrical isolation is obtained within 60 s [25]. These data have been confirmed in the CIRCA-DOSE study: no difference in 1-year efficacy between the 2-min freeze procedure and the 4-min freeze procedure [26]. The COMPARE-CRYO study has recently shown no differences in terms of atrial arrhythmias recurrence between the two commercial cryoballoons currently available [27].

Three prospective RCTs have compared RFA and CBA in PAF patients: these two approaches are similar in terms of efficacy and safety [26,28,29]. In the FIRE and ICE trial, 762 patients were randomized to CBA or RFA. In an intention-to-threat analysis, the CBA group had fewer events during follow-up, but it was associated with higher radiation exposure. Moreover, CBA patients have fewer PV reconnections, but a contact force catheter was used in less than one third of the patients in the RFA group [29]. In Table 1, we summarized principal works that compare RFA vs. CBA in terms of study type, population, results, and safety.

### 3.2. Emerging Alternative Energy Source for Catheter Ablation

Pulsed electrical field ablation (PFA) has emerged as a promising novel technique for cardiac ablation [37]. By applying local electrical fields, PFA induces a modification of the transmembrane cellular potential, which leads to cellular permeability and disruption by creating nanopores on the lipidic membrane layer. One of the unique features of PFA is its tissue selectivity, since cardiomyocytes are the most vulnerable cells to pulsed electrical fields [38,39]. When the applied electric field is optimized to reach the cell-specific critical threshold, cells are unable to recover homeostasis, and cell death occurs, the so-called irreversible electroporation. Cellular effects depend on the magnitude, duration, and waveform characteristics of the applied electrical field. Conversely to more traditional RFA or CBA, PFA is considered a nonthermal energy source, since the mechanism of cell death induction is non-dependent on thermal processes. However, when any electric field is applied to a cell, heating occurs, and any increase in electric field strengths may increase the risk for thermal heat generation and, consequently, shift to thermal effect. For this reason, any combination of PFA parameters and waveform may have its own intensity of effects and should be tested for its clinical safety and efficacy.

Numerous different PFA platforms and devices have been recently designed [37]. At the present time, the pentaspline catheter Farapulse has been shown in large clinical studies to be safe and non-inferior to traditional energy sources in a randomized trial [40]. The pentaspline PFA catheter showed excellent procedural success with acute PVI rates of 99.9% and durability at follow-up of 97–100%. Excellent procedural safety has been reported with serious adverse events occurring in 0–2.5% of patients and, in particular, no energy-specific complications [41,42]. Of note, side effects relevant in thermal energies may not be relevant for PFA, but other unknown side effects may emerge (i.e., acute kidney injury, coronary spasm). Moreover, with the use of PFA, the blanking period could be redefined (from 3 months to 1 month), as shown in a recent work in which only early recurrence occurred in the second or third month after the PFA procedure was associated with a high risk of late recurrence [43].

The promise of (1) highly efficient ablation procedures with short procedural times, (2) creation of transmural and durable lesions, and (3) an enhanced safety profile with minimal to no collateral damage to neighboring structures such as the esophagus or the phrenic nerve makes PFA an ideal energy source for AF catheter ablation.

## 4. Atrial Cardiomyopathy and the Role of Imaging in Risk Stratification

AF is a complex atrial arrhythmia, not only characterized by various clinical presentations but also by several different pathophysiological substrates [18,44]. PAF is usually characterized by increased automaticity or triggered activity, mainly originating in PVs, whereas no overt electrical atrial disease can be detected. On the opposite, persistent AF (PsAF) can present with an advanced electrical atrial disease. In this case, mechanisms underlying the fibrillatory activity are not only increased automaticity and triggered activity but also, and mainly, micro- and macro-reentry resulting in focal impulses, rotors, and multiple wavelets perpetuating AF. Some PAFs can present a broad area of severe electrical disease, even though no overt electrical atrial disease can be detected in many PsAF. For these reasons, there is no univocal AF type, but there are several AFs, and appropriate management of the different AFs still represents a challenge. Therefore, a characterization of the underlying atrial disease, beyond the clinical distinction between paroxysmal and persistent forms, could be needed. Considering several aspects in a “holistic” fashion may allow an early identification of possible diseased atrial tissue.

According to a recent position paper from a multisociety expert panel, the term atrial cardiomyopathy refers to “any complex of structural, architectural, contractile, or electrophysiological changes affecting the atria with the potential to produce clinically relevant manifestation” [44]. A four class descriptive pathological classification has been proposed: (I) principally cardiomyocyte changes; (II) principally fibrotic changes; (III) combined cardiomyopathy pathology and fibrotic changes; (IV) primarily non-collagen infiltration (with or without cardiomyocyte changes). These alterations might be secondary to ventricular or valvular disorders but might also be a primary atrial disorder.

**Figure 3:**
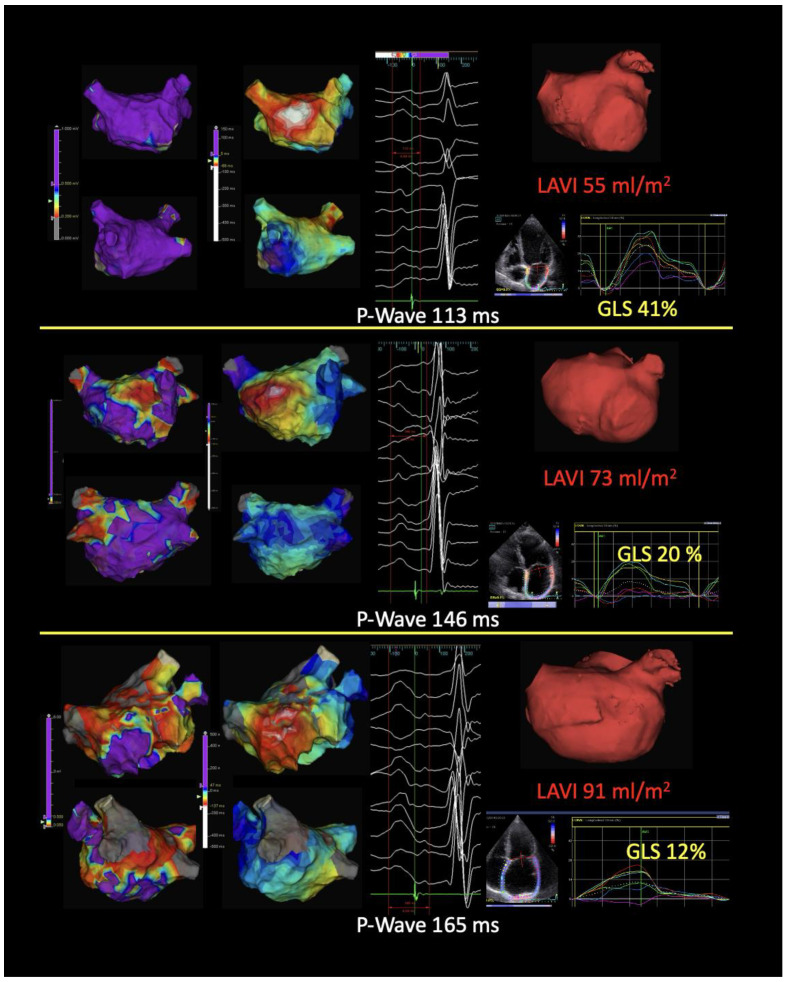
A multimodal approach may contribute to detection of left atrial low voltage area. In these examples from the left to the right: voltage map during sinus rhythm (cutoff < 0.5 mV) and activation map performed with electroanatomical mapping (Ensite SJM), P wave duration, LA dimension detected by CT scan, and deformation detected by echo strain. On top, a case of normal voltage with normal conduction; the P wave is not prolonged, and deformation is normal in this case. In the middle, a case of prolonged P wave, LA enlargement at the CT scan, and impaired strain with moderate low voltage. On the bottom, severely dilated LA, P wave prolongation, and a severely impaired LA strain with a severe degree of low voltage area.

Multimodality cardiac imaging plays an important role in the management of patients who are candidates for AF CA. It allows for the definition of cardiac chamber anatomy and, potentially, of the atrial arrhythmogenic substrates, helping in some cases to guide the ablation strategy and enhance procedural efficacy (Figure 3).

### 4.1. Echocardiography

Transthoracic echocardiography (TTE) is frequently the first-line imaging modality in patients with AF. Standard TTE makes a good estimation of cardiac anatomy, left ventricular systolic and diastolic function, chamber sizes, and valvular function. Several of these parameters, including left atrial (LA) enlargement and left ventricular systolic dysfunction, have been strongly correlated with thromboembolic risk and AF recurrence [45,46]. LA deformation assessed by strain analysis with speckle tracking measures the three phases of atrial function: reservoir, conduit, and booster pump. It has been shown that functional alterations, indicated by a reduction of LA strain, are correlated with atrial fibrosis. The detection of myocardial deformation abnormalities via atrial strain serves as a marker for AF substrate and is associated with the new onset of AF, even in patients with normal atrial volume [47,48,49].

LA thrombus represents an absolute contraindication to CA. An expert panel recommends periprocedural thrombus exclusion in high-risk patients (CHA_2_DS_2_-VASc Score > 1 or presence of high-risk features) and in patients who have not received uninterrupted anticoagulation therapy for at least three weeks [50,51]. About 90% of LA thrombus in non-valvular AF arise in the left atrial appendage (LAA) [52,53]. Transesophageal echocardiography (TOE) can detect atrial thrombus with a sensitivity of 93% to 100% and a specificity of 99% to 100% [54,55]. Moreover, TOE provides important information about LAA contractility dysfunction (defined as LAA emptying velocity < 60 cm/s), spontaneous echocontrast (an independent risk factor for stroke) [56,57] and LAA morphology (e.g., chicken wing, wind-sock, cauliflower, cactus-like), which has been correlated with different stroke risk profiles in some case studies [58,59]. Intracardiac echocardiography (ICE), which offers real-time imaging during procedures, has become increasingly popular and is now considered a standard tool during CA procedures. By assisting the operator during key moments, such as transseptal puncture, ICE enhances the safety of the procedure and reduces the risk of complications [60,61,62]. Furthermore, upcoming advancements in ICE technology will enable 3D real-time imaging, offering volumetric data that can be integrated with electro-anatomical mapping systems, potentially reducing procedure time and the need for extensive catheter manipulation [63]. Lastly, ICE serves as an alternative to transesophageal echocardiography (TEE) for thrombus detection in patients who cannot tolerate TEE, have contraindications, or present with ambiguous findings [60]. The primary drawbacks of ICE are its high cost and the need for a second operator or multitasking during its use.

### 4.2. Cardiac Magnetic Resonance

**Figure 4:**
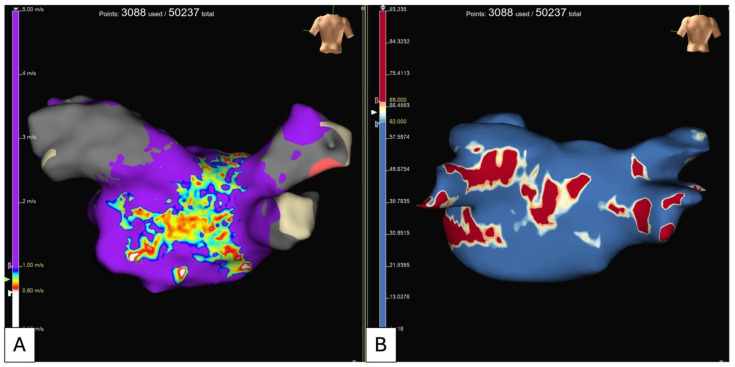
Pre-ablation voltage map of the left atrium with a posterior wall low voltage area (panel **A**). Post-processing CMR images 3D colorimetric map with ADAS 3D software (panel **B**). According to the software, the red areas relate to atrial fibrosis at the level of the posterior wall in this case, matching the low voltage area shown in panel **A**.

Cardiac magnetic resonance (CMR) is a radiation-free and multiplanar technique with high spatial resolution. LA volume measurement in cine sequences is the most accurate technique for atrial volume assessment [64]. Moreover, CMR with angiography offers detailed information on the anatomy of the LA and PVs. Tissue characterization might play an important role in AF CA. In the setting of an AF patient, late gadolinium enhancement (LGE) respiratory-navigated sequences can detect atrial fibrosis. The multicenter prospective trial DECAAF I evaluated the impact of atrial fibrosis on CA outcomes. The authors divided the patients into four categories according to the degree of atrial LGE: stage 1 (atrial fibrosis burden < 10%), stage 2 (10–20%), stage 3 (20–30%), and stage 4 (>30%). They demonstrated that the cumulative incidence of AF recurrence was proportional to the degree of atrial fibrosis (15% for stage 1, 36% for stage 2, 46% for stage 3, and 69% for stage 4) [65]. LGE in LA has also been correlated with LV dysfunction, LAA thrombus, and stroke risk [66,67,68]. Several software options are available for post-processing analysis and LGE quantification, such as ADAS 3D LA (Galgo Medical) [69] (Figure 4).

### 4.3. Computed Tomography

Cardiac computed tomography (CT) is the gold standard for anatomical assessment [70]. Advances in technology have led to improvements in temporal and spatial resolution, artifact reduction, and eventually in image quality. The current CT scan allows for a reduction in the acquisition time and the radiation dose. Cardiac CT allows for the evaluation of the LA wall thickness (WT). Higher WT has been correlated with non-transmural lesions, which, along with non-contiguous lesions, is a major determinant of PV reconnection and AF recurrences after CA. The image data set can be computed by post-processing software (e.g., ADAS 3D, Galgo medical), creating a 3D colorimetric map of LAWT. The map is transferred into the navigation system, allowing the operator to deliver the therapy according to the WT (LAWT-guided pulmonary vein ablation), thereby enhancing CA efficacy and outcomes [71].

In recent years, there has been growing interest about the role of epicardial adipose tissue (EAT), which is the visceral fat located between the myocardium and the visceral pericardium. EAT seems to promote AF producing structural and electrical remodeling of the LA through infiltration on the LA wall and indirectly producing pro-inflammatory and pro-fibrotic mediators [72]. Cardiac CT allows quantification of EAT and detection of fat infiltration of atrial tissue. The amount of fat infiltration has been correlated to AF burden and AF recurrence after CA [73,74]. Evaluation of atrial function, atrial fibrosis quantification, and identification of EAT may give clinically relevant insights into atrial structural changes and atrial cardiomyopathy.

## 5. Beyond Pulmonary Vein Isolation: Ablation of Persistent Atrial Fibrillation

PVI represents the cornerstone of any AF CA and is very effective for rhythm control of PAF but less successful in patients with PsAF. The reported variable success rates after PVI are due to several reasons (e.g., lesions, transmurality, different stages of the disease, and disease progression). Onset and maintenance of PsAF might depend on mechanisms and structures outside the PVs; thus, PVI alone may not be the correct answer to every AF. Despite the numerous improvements in the field, identification of the optimal lesion set beyond PVI for the individual patient remains a still unresolved and crucial point.

Several ablative approaches in addition to PVI have been investigated, with different outcomes and fortunes, which basically consist of (1) extra PV trigger elimination, (2) electroanatomical substrate modification, or (3) empirical anatomical “compartmentalization” (Cox-maze-like) strategies. These strategies may overlap in certain aspects, sharing similar ablation targets or lesion sets, in addition to PVI.

### 5.1. Extra PV Trigger Elimination

**Figure 5:**
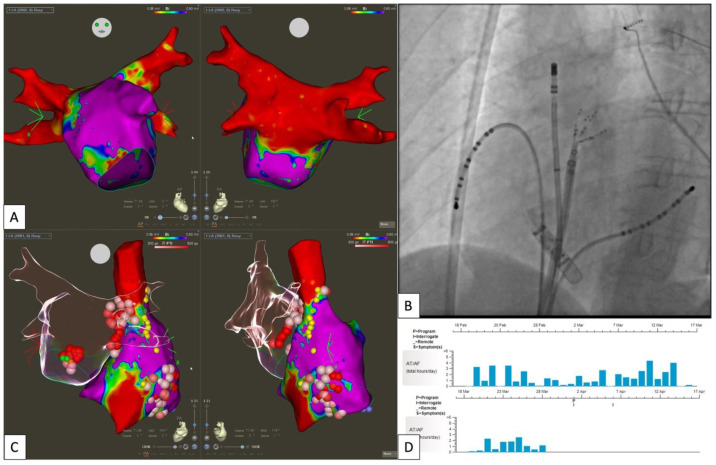
Recurrence of highly symptomatic paroxysmal AF after previous cryoablation (PVI alone) and empirical posterior wall isolation on a second procedure. At the time of the third procedure, the initial map shows persistent PVI and PWI (panel **A**). Drug challenging with high-dose isoproterenol is performed. Several multipolar catheters are placed in strategic positions to map extra PV triggers (in this case at the crista terminalis, SVC, LA anterior wall, and coronary sinus as shown in panel **B**). Biatrial ablation of extra PV (panel **C**). On panel **D** at the upper part, arrhythmia burden after the second procedure, on the bottom after extra PV trigger ablation as detected by implanted loop recorder.

Structures such as the superior vena cava (SVC), LA posterior wall (PW), LAA, ligament of Marshall, and others might be sources of AF initiation and maintenance in resistant cases despite PVI. Independently from the additional target structure, focus identification may be superior to an empirical ablation strategy. Intraprocedural drug challenge with high-dose isoproterenol infusion and eventually adenosine administration may help to identify possible extra PV triggers, thus enhancing the outcome [75] (Figure 5). Although empirical ablation of extra PV foci may be beneficial for selected patients, the overall additive success is controversial and still suboptimal. Consequently, this strategy should be evaluated on a case-by-case basis, especially for patients with identifiable triggers.

**Figure 6:**
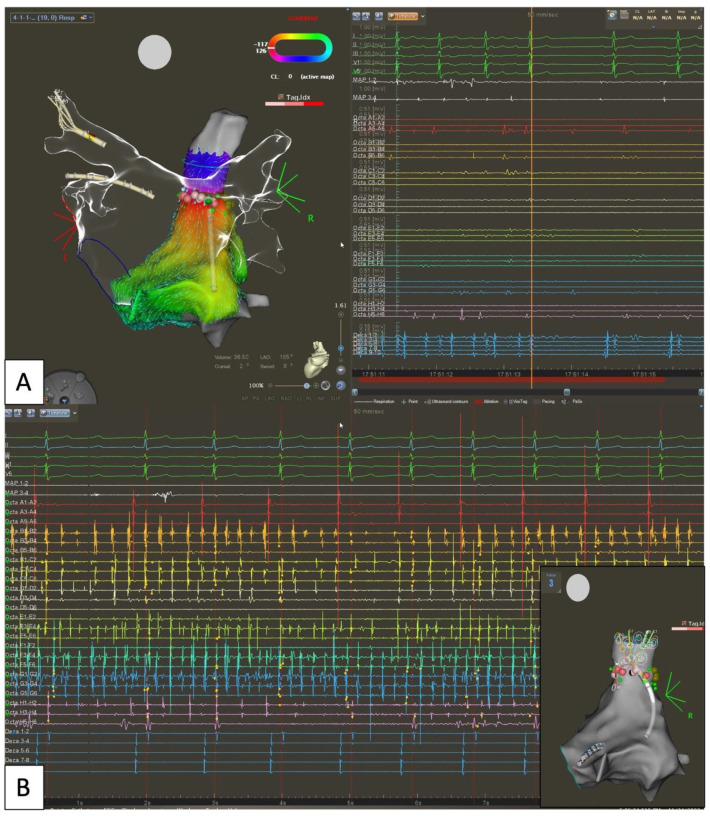
Extra PV trigger elimination—During a de novo RF ablation procedure, spontaneous initiation of sustained AF arises from the superior vena cava. Circumferential SVC ablation results in sinus rhythm restoration (panel **A**), while AF persists confined to the SVC for the whole observation time (panel **B**).

Of note, in the specific case of SVC isolation, monitoring of sinus node and phrenic nerve functionality is essential to avoid collateral injury (Figure 6). Recently, focal PFA has been shown to be effective and safe in consecutive patients undergoing SVC isolation [76].

LAPW, sharing a common embryological origin with PVs, could be an additional source of sustained arrhythmias; thus, it might be a potential additional ablative target. Due to epicardial structures, namely the septopulmonary bundle connecting the so-called LA dome to the PW, which may prevent line of block, durable PW isolation (PWI) might be technically challenging, especially with traditional energy sources. The CAPLA randomized trial showed no significant difference in 1-year freedom from atrial arrhythmias for patients with PsAF undergoing additional PWIs compared to those receiving PVI only [77]. Moreover, esophagus proximity may raise concern about the risk of a life-threatening complication as atrioesophageal fistula [78]. The advent of a non-thermal energy source may overcome the risk of esophageal injury. The pentaspline Farawave catheter has been shown to be well suited for the purpose of PW ablation. However, the additive value of PWI does not seem to be a matter of energy source. A retrospective subanalysis of the MANIFEST-PF trial failed to show additional value of PWI to PVI with Farapulse PFA [79].

**Figure 7:**
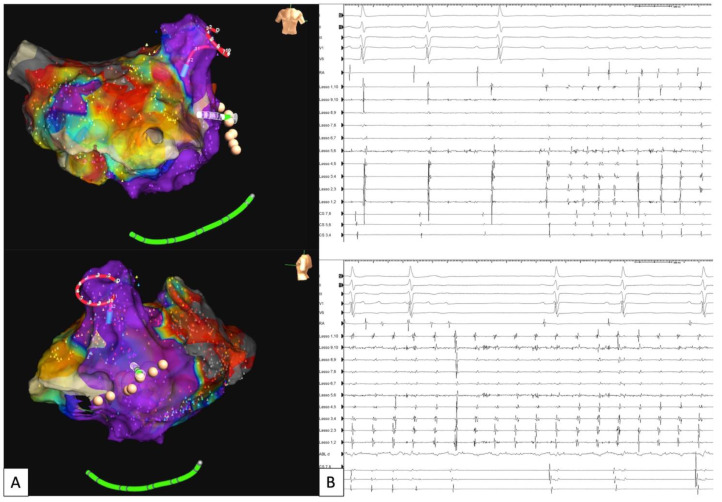
Recurrence of symptomatic persistent atrial fibrillation after previous PVI with roof and anterior mitral lines. panel **A** On sinus rhythm, a three-dimensional electro-anatomical map of the left atrium shows persistent PV isolation and a low voltage (cutoff < 0.5 mV) area on the posterior and on the anterior LA wall. During repeated adenosine applications, evidence of left atrial appendage triggering atrial fibrillation (panel **B**). Linear ablation of the lateral mitral isthmus and conversion to sinus rhythm, while atrial fibrillation persists in the electrically isolated left atrial appendage (decapolar catheter). Modified from Madaffari A. et al. [81] Left Atrial Appendage Electrical Isolation for Persistent Atrial Fibrillation.

LAA electrical isolation (LAAEI) has been associated with improvement in freedom from AF, particularly in those cases where arrhythmias originating from the LAA are induced during the procedure. Two major concerns must be considered: (1) the risk of cardiac perforation due to a thin atrial wall in case of ablation deep into the LAA, (2) an augmented rate of thromboembolic events in case of extensive ablation and wide LAAEI, despite uninterrupted oral anticoagulation therapy. The additional benefit of empirical LAAEI remains controversial and should therefore be limited to a highly selective group of patients, particularly those with potential evidence of arrhythmia originating and/or being maintained within the LAA [80,81] (Figure 7). For the high incidence of LAA thrombi reported after persistent LAAEI, LAA occlusion should be considered [82].

### 5.2. Electroanatomical Substrate Modification (Low Voltage and Complex Fractionated Electrograms Ablation)

Neither linear nor ablation of complex fractionated electrograms (CFE) have shown better results than PVI only. A landmark trial, the STAR-AF II, did show no benefit of additional substrate modification compared to PVI alone for PsAF ablation [83]. Notably, (1) the reported rate of bidirectional block after linear ablation in the trial was only 74%, and (2) the underlying atrial electrical substrate has not been reported. For instance, in a series of unselected patients, we reported successful bidirectional block after LA linear ablation in more than 90% of cases (data presented at EHRA Congress 2015 [84]). A substrate-tailored approach has gained importance in recent years, emphasizing the need for better electrical substrate characterization to guide patient-specific strategies. Low voltage (LVG) ablation aims to deliver personalized therapy by aligning with the patient’s existing electrical disease pattern. This may involve targeted ablation of small areas or larger regions to isolate tissue that shows signs of electrical disease and could become arrhythmogenic. Recent trials suggest better outcomes with LVG ablation combined with PVI [85,86]. Electrophysiological substrate characterization, particularly pre-procedural identification of LA LVG, has become increasingly relevant in stratifying arrhythmogenic risk. However, further investigation is needed, as evidence suggests that electrical and structural remodeling may reverse after restoring sinus rhythm, indicating that the non-PV substrate might be partially reversible [87].

While targeting LVG during sinus or paced rhythm may offer reproducibility and operator independence, CFE ablation during active AF has gained attention with the introduction of artificial intelligence. A machine learning algorithm has been developed to identify spatiotemporal dispersion during ongoing AF. The multicenter TAILORED-AF trial by Deisenhofer et al. showed better outcomes with AI-detected spatiotemporal dispersion ablation in addition to PVI for persistent and long-persistent AF [88]. However, both LVG and CFE ablation strategies could favor the creation of new proarrhythmic electrical substrates by leaving electrical gaps and incomplete linear ablation, as we already learned from an old lesson [89]. Achieving a durable block of any attempted linear ablation is mandatory in order to avoid any potential electrical isthmus for macro-reentrant arrhythmias, which could enhance electroanatomical strategies. Further randomized studies are required to establish the value of LVG-based ablation and the role of artificial intelligence in this field.

### 5.3. Empirical Anatomical “Compartmentalization” (Cox-Maze-like Strategy) and Role of Epicardial Connections

**Figure 8:**
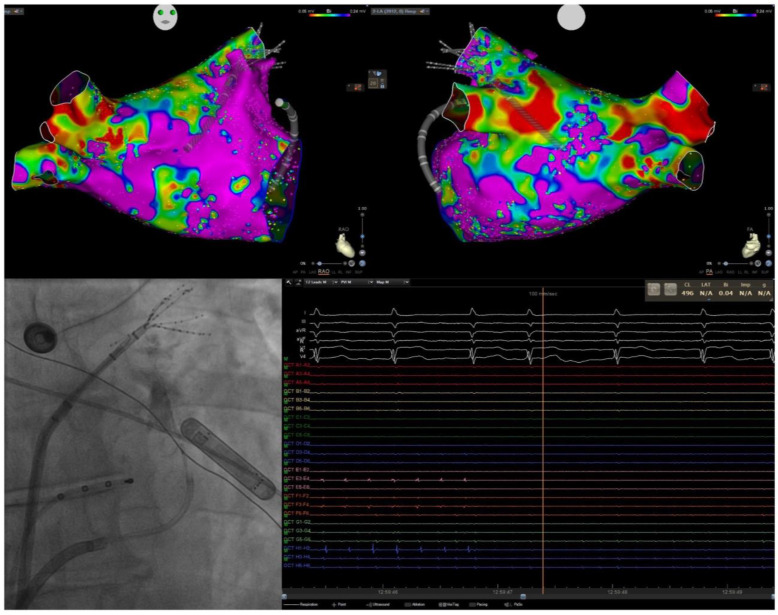
First redo ablation of persistent AF after PVI. The initial voltage map during AF shows severe low voltage with reconnection of the left pulmonary veins (upper panel). During VOM EI, LPV isolation occurs (Octaray catheter in the LSPV—lower panel).

For a decade, additional LA linear ablation lost favor following the results of the STAR-AF II trial. The technical challenge of achieving lines with transmural and durable block notably leaves the door open for future macro-reentrant flutter. Along with improvement in the mapping system with more detailed signal analysis and deeper understanding of arrhythmia mechanisms, attention focused more and more on relevant anatomical structures, which may preclude a line of block. Seminal anatomical works could elucidate the role of epicardial structures such as the vein of Marshall (VOM), the Bachmann’s bundle, and the septopulmonary bundle [90,91]. These structures may serve as an epicardial bridge, preventing transmural line of block. The relevance of epicardial connections in preventing PVs and PW isolation has been largely shown as well [92] (Figure 8).

Several studies have highlighted the ligament of Marshall as an arrhythmogenic substrate, particularly for achieving complete block of a lateral mitral isthmus (MI) line [93,94]. Even with endocardial block, the ligament may serve as an epicardial bridge for sustained macro-reentrant flutter. Similarly, the Bachmann’s bundle can hinder transmural block of an anterior MI line, potentially leading to biatrial macro-reentrant flutter [95,96,97]. Targeting these structures during ablation may improve outcomes. Baez-Escudero et al. first described a method targeting the VOM with retrograde ethanol infusion (VOMEI) [98]. Large trials, including VOMEI, have shown better results with empirical linear ablation compared to PVI alone [99]. A substudy of the VENUS trial suggested that the line of block, rather than the ethanol infusion itself, improves outcomes [100]. Given this evidence, VOMEI is recommended when attempting a lateral MI line to facilitate block formation.

The Bordeaux group has proposed a comprehensive anatomical ablation strategy, a sort of adjusted “Cox-Maze” procedure, the “Marshall-PLAN”, which includes VOMEI to facilitate lateral MI block, wide antral PVI, a roof line, an MI line from the left inferior PV to the mitral annulus, and a cavotricuspid isthmus (CTI) line [101,102]. This approach has shown a 72% freedom from AF/atrial tachycardia recurrence at 12 months for persistent AF patients, rising to 89% after a second procedure. Recently, the PROMPT-AF trial demonstrated that linear ablation combined with VOMEI and PVI significantly improved freedom from atrial arrhythmias compared to PVI alone [103].

**Figure 9:**
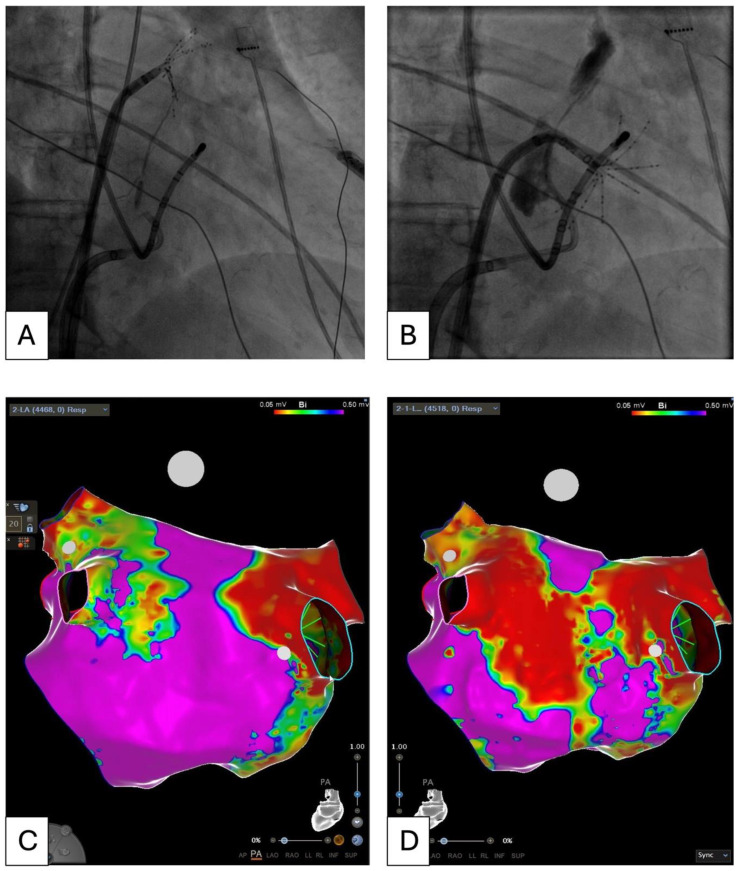
Vein of Marshall ethanol infusion for persistent atrial fibrillation ablation. Fluoroscopic images (right anterior oblique 30°) with VOM visualization after balloon inflation and contrast medium injection (panel **A**); staining lesion along the entire VOM course after ethanol infusion (panel **B**). 3D EAM from a posterior view with voltage map pre (panel **C**) and post VOMEI resulting in left inferior pulmonary vein isolation and partially posterior wall involvement (panel **D**).

However, variability in VOM branching can impact the LVG area resulting from VOMEI, affecting its size and location depending on the arborization pattern (Figure 9). Additionally, some patients may experience arrhythmia recurrence due to scar-related arrhythmias after this empirical linear lesion set [102]. Electrophysiologists often face the dilemma of whether to perform VOMEI and a lateral MI line, ablating an area that typically shows no apparent electrical disease, or to opt for a substrate-tailored ablation, which generally focuses on the LA anterior wall. Anterior MI line ablation is typically not preferred due to evidence supporting VOM; however, it may be the preferred approach in the presence of an extensive scar on the anterior wall, forming a substrate for both perimitral macro-reentry and scar-related micro-reentry arrhythmias. LVG could also be proarrhythmogenic, leading to a choice between approaches. Both ablation lines face the challenge of achieving transmural complete block, with the ligament of Marshall and Bachmann’s bundle acting as epicardial bridges for the lateral and anterior MI lines, respectively. Randomized data are still needed. Performing both complete lateral and anterior MI lines along with PVI and a roof line will result in a wide LAAEI. Finally, it is the author’s opinion that isolating the anterior aspect of the left PVs from the LA side (rather than the PV side) may help facilitate lateral MI line block by partially affecting the ligament of Marshall.

### 5.4. Pulsed Field for Extra PV Trigger Ablation

Based on a strategy of atrial electrical compartmentalization, the Cox-Maze procedure is the most successful in terms of arrhythmia freedom for PsAF ablation so far [104]. Reproducibility of a catheter-based Cox-maze-like lesion set is still suboptimal, due to technical and energy limitations. The reported rate of incomplete, non-transmural, non-durable ablation lines is still high with traditional techniques, even combined with VOMEI. The initial enthusiasm for PFA in shifting the landscape of AF CA and PVI could not yet be translated into a more extensive lesion set strategy.

**Figure 10:**
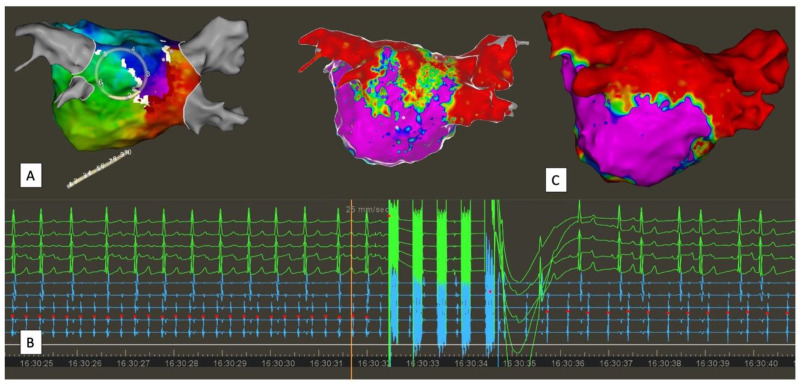
PFA for atypical flutter ablation—Persistent flutter after PVI. A map of the ongoing flutter performed with a multipolar catheter (Octaray–CARTO) shows a scar-related atypical flutter localized to the LA posterior wall (panel **A**) left: activation map; right: voltage map during ongoing flutter). The Farapulse PFA catheter is visualized in this case as a circular catheter by the CARTO mapping system. A single PFA application at the critical isthmus, as shown, terminates the arrhythmia with restoration of sinus rhythm (panel **B**). Complete posterior wall isolation with additional application is successfully performed (panel **C**).

PFA Farapulse has been tested in clinical studies for linear ablation as well as for atypical left atrial flutter ablation. PW ablation with the pentaspline PFA catheter can be safely and efficiently performed, with a high durability observed during redo procedures [105]. The pentaspline PFA catheter is particularly well suited for the application of a roof line and for LAPW isolation (Figure 10). However, the added value of durable PWI with PFA for the treatment of AF has still to be evaluated. On the other hand, the same catheter is less effective for ablation of the MI and the anterior line, for which additional RF ablation may be required to achieve bidirectional block [106]. Particularly, PFA energy for ablation of the lateral MI line may cause coronary vasospasm because of the proximity to the circumflex artery. Applying a more anterior MI line may lead to a large area of scar tissue, which presents the downside of making electrogram interpretation difficult, if not impossible—a well-documented effect with PFA.

As a valid alternative, a novel focal lattice tip catheter with a large footprint allows for toggling between PFA and RF ablation. At the present time, evidence and clinical application of the Sphere-9 catheter combined with the Affera system are limited, but the initial results are very encouraging. In unselected patients, PVI combined with linear ablation can be highly effective, achieving excellent lesion durability upon mandatory remapping after waveform optimization [107,108]. This could mark the beginning of a new era in AF therapy and invasive electrophysiology, where the availability of reliable technology to deliver effective and durable treatment will enable meaningful comparison of ablation strategies.

### 5.5. Convergent Hybrid Ablation: Is This Technique Obsolete in the PFA Era?

Endocardial ablation can be combined with minimally invasive thoracoscopic epicardial ablation. This hybrid ablation (HA) for AF involves a Cox-Maze lesion set via a thoracoscopic approach, targeting epicardial substrate (such as the septopulmonary bundle), followed by endocardial ablation to address gaps in the lesion set. In literature are described various surgical approaches and energy used, the timing of the two steps (endocardial and epicardial), the ablation lesion set applied, and the management of the LAA [109]. Among the various surgical approaches described, the convergent technique is one of the most investigated. It is based on PWI through a subxiphoid approach and a vacuum-assisted unipolar RF probe. The multicenter, randomized controlled CONVERGE trial has demonstrated a superiority of HA over endocardial ablation alone. In this trial, the authors showed the superiority of the hybrid approach compared with endocardial CA-only for the treatment of persistent and long-standing PsAF. One hundred and fifty-three patients were randomized 2:1 to hybrid convergent procedure versus endocardial catheter ablation. At 1-year follow-up, freedom from atrial tachyarrhythmias on AADs was achieved in 67.7% (67/99) of patients with hybrid convergent vs. 50.0% (25/50) with catheter ablation (*p* = 0.036) and in 53.5% (53/99) vs. 32.0% (16/50; *p* = 0.0128) off AADs. Moreover, at a long term follow-up (18 months), 74% of patients in the hybrid arm achieved at least 90% AF burden reduction when compared to 55% with endocardial CA only (*p* = 0.0395). Notably, the authors report 8% major adverse events in the hybrid convergent arm (0% on the other) [110]. The advent of PFA is likely to overcome this surgical-related risk. A recent study compared hybrid convergent RF ablation with endocardial ablation alone with PFA, concluding that while both techniques yield similar arrhythmic outcomes, the epicardial approach is associated with higher periprocedural risks [111].

## 6. Future Perspectives and Conclusions

Catheter ablation for atrial fibrillation is a well-established treatment for rhythm control, reducing recurrences and progression of the disease. Ongoing innovations and emerging evidence are broadening its role as first-line therapy. Pulmonary vein isolation represents the cornerstone of any atrial fibrillation catheter ablation. However, outcomes are still suboptimal for persistent or long-standing persistent atrial fibrillation. Despite several improvements in ablation technologies, little progress has been made in understanding the arrhythmia mechanisms. The advent of artificial intelligence might help to enhance procedural success. Whether these technologies will influence the standard treatment strategies remains uncertain. Pulsed field ablation is a new energy modality, holding the promise of highly efficient ablation procedures with short procedural times, the creation of transmural and durable lesions, and an enhanced safety profile. The optimal interventional treatment for persistent atrial fibrillation is still controversial. The advent of new ablation platforms with the possibility of different energy sources might help to deliver more effective and durable lesions and to standardize the lesion set, enabling to meaningfully compare different ablation strategies.

## Figures and Tables

**Table 1:** Radiofrequency versus Cryoballoon for atrial fibrillation ablation.

Study	Study Type	N Patients	Results	Safety
Luik et al. FreezeAF (2015) [28]	Multicenter RCT	159 to RFA, 141 to CBA	Efficacy: CBA was non- inferior to RFA.	5.0% RFA versus 12.2% CBA, *p* = 0.022
Kuck et al. (FIRE AND ICE) 2016 [29]	Multicenter RCT	762 (378 RFA-384 CBA) PAF only	Efficacy: CBA is non-inferior to RFA.	no difference
Boveda et al., 2016 [30]	Multicenter, prospective observational	59 CBA 59 RFA Ps AF	Efficacy: CBA was non-inferior to RFA.	Patients undergoing RFA presented a numerically, but non-significantly, lower complication rate (6.8% vs. 10.2%, *p* = 0.51).
Gunawardene et al., 2016 [31]	RCT	30 CBA (2 gen) 30 RFA PAF only	Efficacy: No difference in early recurrence rates of atrial fibrillation (ERAF).	No difference.
Watanabe et al., 2018 [32]	RCT	24 CBA 25 RFA PAF only	-	CBA may reduce the acute narrowing of the left-sided PVs as compared to RFA ablation.
Buist et al., 2018 [33]	RCT	136 CBA 133 RFA PAF and PsAF	Single procedure freedom from atrial arrhythmias was significantly higher in CBA as compared to RFA (75.2 vs. 57.4%, *p* = 0.013).	No difference.
Andrade et al., 2019 [26] (CIRCA DOSE)	Multicenter RCT	346 (RFA115:CBA 4 min 116:CBA 2 min 115) PAF only	No difference in 1-year efficacy (time to first recurrence and burden re- duction assessed by ILR). Less fluoroscopy time for RFA.	no difference.
Natale et al., 2021 [34]	Retrospective observational	407 RFA 1066 CBA	-	No difference
Bisignani et al., 2022 [35]	Retrospective observational	30 patients undergoing PVI + LA posterior wall isolation (LAPWI) with CBA, 30 patients who underwent PVI + linear ablation (roof and mitral lines) using RFA, 60 patients with PVI alone using CBA, and 60 patients who had PVI alone using RFA PsAF	LAPW ablation in addition to PVI with CBA seems to improve 1-year outcomes in comparison to PVI + linear ablation using RFA and to PVI alone using RFA or CBA.	No difference.
Shi et al., 2022 [36]	RCT	49 RFA 52 CBA PsAF	No difference in AF recurrence, less atrial flutter recurrence documented in the CBA group compared with the RFA group (3.9% vs. 18.0%, *p* = 0.020).	No difference.
